# Yeast culture derived from Jiang-flavor Baijiu distiller's grains enhances immune function and gut health in geese

**DOI:** 10.3389/fvets.2026.1846477

**Published:** 2026-05-22

**Authors:** Pan Tao, Fei Zhang, Yu-jie Li, Xiao-jie Chen, Yi-ming Zuo, Zhen-hong Peng, Wei-kuan Ji, Yan-tao Lv, Yun-mao Huang

**Affiliations:** 1 Guangdong Engineering Technology Research Center of Biosafety and Intelligent Control for Aquatic Animals Diseases, Zhongkai University of Agriculture and Engineering, Guangzhou, China; 2 Road Biological Environmental Protection Technology Co., Ltd., Wuhan, China; 3 Waterfowl Healthy Breeding Engineering Research Center of Guangdong, Zhongkai University of Agriculture and Engineering, Guangzhou, China

**Keywords:** goose, immune performance, intestinal health, JBGC, yeast culture

## Abstract

**Introduction:**

This study evaluated the effects of dietary supplementation with Jiang-flavor Baijiu distiller's grains yeast culture (JBGC) on immune performance and intestinal health in geese.

**Methods:**

A total of 512 one-day-old Magang geese were randomly assigned to four dietary treatments 0% (control), 2%, 6%, or 10% JBGC with eight replicates per treatment and 16 birds per replicate, over a 70-day experimental period.

**Results:**

JBGC supplementation exhibited dose- and time-dependent effects on antioxidant and inflammatory responses. Compared with the control, 10% JBGC significantly reduced serum glutathione peroxidase activity at day 70 and total antioxidant capacity at day 28 (*P* < 0.05), whereas both 6% and 10% JBGC increased hepatic total superoxide dismutase activity (*P* < 0.05). Regarding inflammatory markers, 10% JBGC lowered serum IL-1β at day 70 (*P* < 0.05), and all JBGC-supplemented groups (2%, 6%, and 10%) showed significantly lower IL-8 levels than the control during the later phase (*P* < 0.05). Additionally, 6 and 10% JBGC elevated the anti-inflammatory cytokine IL-10 at day 70 (*P* < 0.05). JBGC also improved intestinal morphology and barrier function: 6 and 10% JBGC increased ileal villus height (*P* < 0.05); 10% JBGC enhanced duodenal length and weight, jejunal weight, and total small intestinal weight; and 6% JBGC upregulated the protein expression of occludin and claudin?5 (*P* < 0.05). Although cecal microbial alpha diversity did not differ significantly, several beneficial bacterial genera showed increasing trends.

**Discussion:**

These findings indicate that JBGC can positively modulate immune and intestinal health in geese by influencing antioxidant status, inflammatory cytokine profiles, intestinal morphology, and barrier integrity.

## 1 Introduction

As the valorization of agricultural by-products gains increasing attention, fermented derivatives—particularly those from distiller's grains—have emerged as cost-effective feedstuffs with considerable untapped nutritional potential (1). Among these, yeast culture derived from Jiang-flavor distiller's grains (JBGC) is a distinctive functional ingredient, enriched not only with high-quality protein (20%−23.5%) but also with bioactive compounds such as acid-soluble proteins, lactic acid, and viable probiotics. These constituents collectively contribute to improving the gastrointestinal microenvironment in livestock (2–4).

Yeast cultures are widely documented to enhance digestion, immunity, and overall performance across various livestock species. In poultry, dietary yeast supplementation improves intestinal morphology, serum antioxidant capacity, and gut microbiota composition (4–7). In pigs, yeast culture promotes nutrient digestibility and growth performance while alleviating hepatic metabolic stress (8–10). Similar benefits are observed in ruminants and aquatic animals, where yeast cultures enhance rumen function, milk yield, and overall productivity (11).

The mechanisms underlying these benefits are increasingly well understood. Yeast cultures act as prebiotics, modulating gut microbial balance by increasing beneficial bacteria (e.g., Lactobacillus, Bifidobacterium) and inhibiting pathogens such as Clostridium perfringens (12–14). Moreover, microbial fermentation of yeast components yields short-chain fatty acids (SCFAs), which enhance intestinal epithelial integrity through upregulation of tight-junction proteins (e.g., occludin, ZO-1) and support villus development (15, 16). These local effects are closely linked to systemic immune modulation, as the gut microbiota plays a central role in shaping host immunity (17).

Despite these advances, significant knowledge gaps remain—particularly in waterfowl. Most existing studies focus on chickens, pigs, or ruminants, leaving species-specific responses in geese poorly characterized. Geese possess unique digestive and immune traits compared with terrestrial poultry, yet the long-term physiological impacts of yeast culture supplementation in this species are scarcely reported. Furthermore, although dose-dependent effects of yeast cultures have been noted in Sichuan white geese (18, 19), the holistic influence of JBGC—with its distinctive compositional profile derived from Jiang-flavor fermentation—on goose intestinal health, systemic immunity, antioxidant capacity, and gut microbiota has not been systematically examined.

Therefore, this study aimed to investigate the effects of JBGC supplementation on key physiological parameters in geese, including immune organ development, hepatic antioxidant function, serum inflammatory markers, intestinal barrier integrity, and gut microbiota composition. The findings provide new insights into species-specific applications of yeast cultures and support the development of functional feeding strategies for waterfowl.

## 2 Materials and methods

### 2.1 Experimental design and diets

A total of 512 healthy 1-day-old Magang geese (mixed sex) were obtained from Shengyi Poultry Breeding Co., Ltd. The geese were randomly allocated to four treatment groups, each with eight replicates and 16 birds per replicate. The groups were defined as follows:

Zero percentage JBGC (Control): fed a basal corn–soybean meal diet without JBGC supplementation.

Experimental groups: fed the same basal diet supplemented with 2%, 6%, or 10% JBGC (provided by Lude Bio-Environmental Protection Technology Co., Ltd.; which is produced by solid-state fermentation of fresh Jiang-flavor Baijiu distiller‘s grains with Saccharomyces cerevisiae; detailed composition in Table 1; JBGC was supplied as a dry powder and was thoroughly mixed into the basal diets before pelleting).

**Table 1:** Composition of JBGC.

Component	Content (%)	Component	Content (%)
Moisture (%)	12.80	Lysine (Lys) (%)	0.28
Crude protein (CP) (%)	22.40	Leucine (Leu) (%)	1.63
Calcium (Ca) (%)	0.45	Proline (Pro) (%)	1.42
Total phosphorus (%)	0.62	Serine (Ser) (%)	0.70
Ether extract (EE) (%)	6.10	Threonine (Thr) (%)	0.57
Crude fiber (CF) (%)	9.80	Aspartic acid (Asp) (%)	1.10
Crude ash (Ash) (%)	7.10	Valine (Val) (%)	0.81
Water-soluble chloride (as NaCl) (%)	0.16	Isoleucine (Ile) (%)	0.65
Acid detergent fiber (ADF) (%)	16.10	Histidine (His) (%)	0.41
Neutral detergent fiber (NDF) (%)	18.70	Total amino acids (oxidative hydrolysis) (%)	15.82
Methionine (Met) (%)	0.29	Acid-soluble protein g/100 g	8.93
Cystine (Cys) (%)	0.24	Small peptides %	8.01
Phenylalanine (Phe) (%)	0.82	Lactic acid %	12.50
Alanine (Ala) (%)	1.33	Nucleotides (%)	0.80
Glycine (Gly) (%)	0.71	Nucleic acids (%)	2.52
Glutamic acid (Glu) (%)	4.46	Mannan oligosaccharides (%)	6.42
Arginine (Arg) (%)	0.40	β-1, 3-D-glucan (%)	4.31

All geese were reared in a floor pen system with *ad libitum* access to feed and fresh water throughout the experimental period. Routine immunization procedures (e.g., for common avian pathogens) were implemented to ensure flock health. The experiment lasted 70 days, covering the key growth stages of Magang geese.

To eliminate nutritional confounding factors, metabolizable energy, crude protein, and other essential nutrient levels were standardized across all groups. Basal diets for each growth stage were formulated according to a corn–soybean meal framework and referenced the recommended nutritional requirements for Magang geese; detailed nutritional compositions are presented in Tables 2–4.

**Table 2:** Diet formulation and nutrient levels for meat geese aged 1-14 days.

Ingredients	Ratio (%)			
	0% JBGC	2% JBGC	6% JBGC	10% JBGC
Corn (grade 2^*^)	51.77	51.18	51.00	50.07
Wheat bran (grade 1)	19.00	18.00	15.00	13.00
Soybean meal (grade 2)	24.40	23.90	23.00	21.90
DL-met (99%)	0.00	2.00	6.00	10.00
Lys-HCl (78%)	0.21	0.21	0.20	0.21
Arg (98%)	0.40	0.43	0.48	0.52
L-Thr (98%)	0.17	0.23	0.23	0.24
Trp (98%)	0.05	0.05	0.07	0.08
Limestone powder (36%)	1.10	1.10	1.02	0.98
Dicalcium phosphate	1.60	1.60	1.70	1.70
Salt	0.30	0.30	0.30	0.30
Premix	1.00	1.00	1.00	1.00
Total	100.00	100.00	100.00	100.00
Nutrient levels				
ME, Mcal/kg	2.90	2.90	2.90	2.90
CP, %	18.26	18.26	18.26	18.26
Ca, %	0.89	0.89	0.89	0.89
P, %	0.72	0.72	0.72	0.72
Lys, %	1.23	1.23	1.23	1.23
Met, %	0.46	0.46	0.46	0.46
Thr, %	0.88	0.88	0.88	0.88
Trp, %	0.26	0.26	0.26	0.26

^*^Grade classifications are based on Chinese National Standards GB/T 17889–1999 (wheat bran) and GB/T 19541–2017 (soybean meal). Grade 1 indicates higher nutritional quality than Grade 2.

**Table 3:** Diet formulation and nutrient levels for meat geese aged 15–42 days.

Ingredients	Ratio (%)			
	0% JBGC	2% JBGC	6% JBGC	10% JBGC
Corn (grade 2^*^)	59.00	59.00	59.00	58.50
Wheat bran (grade 1)	23.20	22.00	19.24	17.30
Soybean meal (grade 2)	12.26	11.45	10.00	8.30
DL-met (99%)	0.00	2.00	6.00	10.00
Lys-HCl (78%)	0.26	0.26	0.26	0.26
Arg (98%)	0.59	0.62	0.68	0.74
L-Thr (98%)	0.30	0.33	0.40	0.47
Trp (98%)	0.28	0.29	0.30	0.33
Limestone powder (36%)	0.12	0.13	0.14	0.16
Dicalcium phosphate	0.99	0.91	0.89	0.80
Salt	1.70	1.71	1.79	1.84
Premix	0.30	0.30	0.30	0.30
Total	1.00	1.00	1.00	1.00
Ingredients	100.00	100.00	100.00	100.00
Nutrient levels				
ME, Mcal/kg	2.85	2.85	2.85	2.85
CP, %	15.50	15.50	15.50	15.50
Ca, %	0.83	0.83	0.83	0.83
P, %	0.72	0.72	0.72	0.72
Lys, %	1.07	1.07	1.07	1.07
Met, %	0.45	0.45	0.45	0.45
Thr, %	0.76	0.77	0.77	0.77
Trp, %	0.27	0.27	0.27	0.27

^*^Grade classifications are based on Chinese National Standards GB/T 17889–1999 (wheat bran) and GB/T 19541–2017 (soybean meal). Grade 1 indicates higher nutritional quality than Grade 2.

**Table 4:** Diet formulation and nutrient levels for meat geese aged 43–70 days.

Ingredients	Ratio (%)			
	0% JBGC	2% JBGC	6% JBGC	10% JBGC
Corn (grade 2^*^)	62.20	62.00	61.40	61.54
Wheat bran (grade 1)	18.28	17.13	15.20	12.50
Soybean meal (grade 2)	14.00	13.30	11.70	10.10
DL-met (99%)	0.00	2.00	6.00	10.00
Lys-HCl (78%)	0.26	0.26	0.26	0.26
Arg (98%)	0.66	0.68	0.73	0.80
L-Thr (98%)	0.31	0.33	0.40	0.47
Trp (98%)	0.33	0.34	0.36	0.37
Limestone powder (36%)	0.12	0.13	0.14	0.16
Dicalcium phosphate	0.65	0.62	0.54	0.50
Salt	1.89	1.91	1.97	2.00
Premix	0.30	0.30	0.30	0.30
Total	1.00	1.00	1.00	1.00
Ingredients	100.00	100.00	100.00	100.00
Nutrient levels				
ME, Mcal/kg	2.91	2.91	2.91	2.91
CP, %	15.90	15.90	15.90	15.90
Ca, %	0.75	0.75	0.75	0.75
P, %	0.73	0.73	0.73	0.73
Lys, %	1.15	1.15	1.15	1.15
Met, %	0.46	0.46	0.46	0.46
Thr, %	0.83	0.83	0.83	0.83
Trp, %	0.27	0.27	0.27	0.28

^*^Grade classifications are based on Chinese National Standards GB/T 17889–1999 (wheat bran) and GB/T 19541–2017 (soybean meal). Grade 1 indicates higher nutritional quality than Grade 2.

### 2.2 Analysis of serum antioxidant and immune-related indicators

At 14, 28, and 70 days of age, geese were weighed after fasting. Two geese per replicate were selected, and 5 ml of blood was collected from the wing vein. Blood samples were centrifuged at 3,000 × g for 10 min at room temperature; sera were separated, aliquoted, and stored at −80 °C.

Commercially available enzyme-linked immunosorbent assay (ELISA) kits (Nanjing Jiancheng Bioengineering Institute, Nanjing, China) were used to determine serum antioxidant indices, including total superoxide dismutase (T-SOD) and glutathione peroxidase (GPx) activities, as well as total antioxidant capacity (T-AOC) and malondialdehyde (MDA) concentration.

A separate set of ELISA kits (Huabodeyi Biological Science and Technology Co., Ltd., Beijing, China) was used to measure serum concentrations of anti-inflammatory and pro-inflammatory cytokines (IL-1β, IL-6, IL-8, IL-10, TNF-α, IFN-γ) and immunoglobulins (IgG, IgA, IgM).

### 2.3 Histomorphological analysis of the ileum

After slaughter at 70 days of age, the length and weight of the small intestine were recorded. A 1 cm segment from the mid-jejunum was collected and fixed in 10% formalin. Ileal tissue samples fixed in 10% formalin were sectioned, stained with hematoxylin and eosin (HE), and examined morphologically. Villus height, villus width, crypt depth, and muscular layer thickness were measured using ImageJ software; the villus height-to-crypt depth ratio (V/C ratio) was calculated.

### 2.4 RNA isolation and gene expression analysis in ileal tissue

The mid-ileum segment was collected, gently flushed with sterile PBS (pH 7.4) to remove luminal contents, immediately snap-frozen in liquid nitrogen, and stored at −80 °C until analysis. Total RNA was extracted from ileal tissues using TRIzol™ Reagent (Invitrogen™, Cat. No. 15596018CN) according to the manufacturer's instructions. Reverse transcription was performed with the TaKaRa PrimeScript™ RT Master Mix (TaKaRa, Cat. No. RR036A). Primers were designed using Primer 5.0 software (Table 5) and synthesized by Sangon Biotech Co., Ltd. (Shanghai, China). Quantitative real-time PCR (qRT-PCR) was carried out in 20 μL reactions containing 10 μL 2 × SYBR Premix EX Taq, 0.8 μL each of forward and reverse primer (10 mM), 1 μL cDNA, and 7.4 μL nuclease-free water, using a QuantStudio 7 Flex Real-Time PCR System (Thermo Fisher Scientific, Cat. No. 4485690). Relative gene expression was calculated using the 2^−Δ*ΔCT*^ method, with β-actin as the internal reference.

**Table 5:** Sequences of primers used for quantitative real-time PCR.

Gene	Accession no.	Sequence (5^′^ → 3^′^)
β-actin	EF687232	F: GCTATGTCGCCCTGGATTT
		R: GGATGCCACAGGACTCCATAC
IL-1β	JX173025	F: CAGCTTCACCTGCAGTTCC
		R: TGGTCCAGGTAGACGGTGAT
IL-6	JX173026	F: AGATGGTGATAAATCCTGATGA
		R: CGGTTTTCTCCATAAATGAAGT
IL-8	JX173027	F: ATGAACGGCAAACTTGGGGCT
		R: GCCAGAATTGCCTTTACGATCAG
IL-10	JX173028	F: GCTGAGGATAAAGTTCGAGG
		R: CCGAAGGTCCCCTTAAATTC
TNF-α	JX173024	F: TGTGTATGTGCAGCAACCCGTAGT
		R: GGCATTGCAATTTGGACAGAAGT
Claudin-1	XM_013186712.3	F: CTCGTCGTCGCCGGTGT
		R: GCATCGGCGGCGACACC
Claudin-3	XM_038224373.1	F: CTC GCC CTT GTG GTC ATT
		R: GAG GCT GGC ATC TCC TTA
Claudin-5	XM_013176600.3	F: GTCCCGCTCTGCTGGTTC
		R: CCCTATCTCCCGCTTCTGG
Occludin	XM_013186404.3	F: CGCCACGTTCTTCACCCACTC
		R: CTCATCTGCTTCTTCGCCCACA
ZO-1	XM_013168266.3	F: CTTCAGGTGTTTCTCTTCCTCCTC
		R: CTGTGGTTTCATGGCTGGATC

### 2.5 Western blot analysis

Western blot analysis was performed to determine the protein expression levels of barrier function-related tight junction proteins (occludin, claudin-1, claudin-3, claudin-5). Ileal tissues were homogenized on ice in lysis buffer containing PMSF. Following centrifugation, protein supernatants were collected, subjected to electrophoresis, and transferred to PVDF membranes. Membranes were blocked with 5% non-fat milk or BSA, incubated overnight at 4 °C with primary antibodies (1:1,000 dilution), and then incubated with corresponding HRP-conjugated secondary antibodies (1:5,000 dilution) at room temperature. After thorough washing, protein bands were visualized using ECL substrate and quantified by grayscale analysis using ImageJ software.

### 2.6 Microbial community analysis

Cecal contents were collected, immediately frozen in liquid nitrogen, and stored at −80 °C until analysis. Total genomic DNA was extracted using the OMEGA Soil DNA Kit (M5635-02; Omega Bio-Tek, Norcross, GA, USA) according to the manufacturer's instructions and stored at −20 °C. DNA quantity and quality were assessed using a NanoDrop NC2000 spectrophotometer (Thermo Fisher Scientific, Waltham, MA, USA) and agarose gel electrophoresis. PCR amplification of the V3–V4 region of the bacterial 16S rRNA gene was performed using the forward primer 338F (5′-ACTCCTACGGGAGGCAGCA-3′) and reverse primer 806R (5′-GGACTACHVGGGTWTCTAAT-3′). Sequencing and bioinformatic analysis were conducted by Personalbio (Shanghai, China).

### 2.7 Statistical analyses

All data are expressed as mean ± standard error of the mean (SEM). Statistical analyses were performed using one-way analysis of variance (ANOVA) with SAS 8.1 software, followed by Duncan's multiple range test. Differences were considered statistically significant at *P* < 0.05.

## 3 Results

### 3.1 Effects of JBGC on antioxidant indices in serum and liver

The effects of JBGC on serum antioxidant parameters are presented in Table 6. JBGC supplementation exerted dose- and time-dependent effects. Serum GPx activity was significantly reduced in the 10% JBGC group compared with all other groups at 70 days of age (*P* < *0.01*), with no significant differences at earlier time points. Serum T-AOC was significantly lower in the 10% JBGC group than in the control at 28 days (*P* < *0.01*). By day 70, however, the 10% JBGC group exhibited significantly higher T-AOC than the other groups (*P* < *0.05*). Serum T-SOD activity differed significantly only at 70 days: the 10% JBGC group showed higher activity than the 2% group (*P* < *0.05*) but did not differ significantly from the control. Serum MDA concentrations remained unaffected across all time points.

**Table 6:** Effects of different levels of JBGC in the diet on blood antioxidant indices of geese.

Indicator	0% JBGC	2% JBGC	6% JBGC	10% JBGC	SEM	*P*-value
T-SOD, U/ml						
Day 14	0.88	0.85	0.93	0.88	0.02	0.40
Day 28	1.50	1.46	1.47	1.43	0.01	0.40
Day 70	1.82^ab^	1.78^b^	1.81^ab^	1.85^a^	0.01	0.04
Gpx, nmol/L						
Day 14	198.09	172.00	189.28	197.67	6.10	0.43
Day 28	198.74	197.74	199.52	196.02	0.76	0.80
Day 70	220.85^a^	221.08^a^	220.99^a^	213.91^b^	1.77	< 0.01
T-AOC, mmol/L						
Day 14	0.20	0.16	0.17	0.16	0.01	0.20
Day 28	0.13^a^	0.13^a^	0.12^a^	0.10^b^	0.01	< 0.01
Day 70	0.14^b^	0.14^b^	0.14^b^	0.17^a^	0.01	0.01
MDA, nmol/ml						
Day 14	4.38	6.46	6.80	5.67	0.54	0.36
Day 28	7.21	7.08	9.33	8.33	0.53	0.58
Day 70	9.75	8.93	6.37	8.36	0.72	0.86

JBGC, Jiang-flavor Baijiu distiller's grains yeast culture; T-SOD, total superoxide dismutase; GPx, glutathione peroxidase; T-AOC, total antioxidant capacity; MDA, malondialdehyde. Values are presented as mean ± SEM (*n* = 8). Superscript letters (a, b) in the same row denote significant difference (*P* < 0.05) by Duncan's test.

Hepatic antioxidant indices are summarized in Table 7. Compared with the control, hepatic T-SOD activity was significantly higher in the 6% and 10% JBGC groups (*P* < *0.05*), whereas no significant difference was observed in the 2% JBGC group. Hepatic T-AOC was significantly lower in the 2% JBGC group than in the control (*P* < *0.05*), with no significant differences in the 6% and 10% groups. Hepatic MDA and GPx concentrations did not differ significantly between any JBGC-supplemented group and the control.

**Table 7:** Effects of different levels of JBGC in the diet on liver antioxidant indices of geese.

Indicator	0% JBGC	2% JBGC	6% JBGC	10% JBGC	SEM	*P*-value
T-SOD (U/ml)	17.12^b^	15.24^b^	23.31^a^	23.39^a^	2.11	< 0.01
T-AOC (mmol/L)	0.25^a^	0.14^b^	0.21^ab^	0.16^ab^	0.02	0.02
MDA (nmol/ml)	0.80	0.60	0.77	0.67	0.05	0.44
Gpx (nmol/L)	63.10	69.01	74.43	88.60	5.45	0.13

JBGC, Jiang-flavor Baijiu distiller's grains yeast culture; T-SOD, total superoxide dismutase; GPx, glutathione peroxidase; T-AOC, total antioxidant capacity; MDA, malondialdehyde. Values are presented as mean ± SEM (*n* = 8). Superscript letters (a, b) in the same row denote significant difference (*P* < 0.05) by Duncan's test.

### 3.2 Effects of JBGC on anti-inflammatory/pro-inflammatory factors in blood

Serum cytokine concentrations are shown in Table 8. JBGC supplementation exerted distinct dose- and time-dependent immunomodulatory effects. At 70 days, the 10% JBGC group exhibited significantly lower IL-1β (*P* < *0.01*) and significantly higher IL-10 (*P* < *0.01*) compared with the control. All JBGC-supplemented groups (2%, 6%, and 10%) consistently suppressed IL-8 at both 28 and 70 days (*P* < *0.01*). At day 70, IL-6 was significantly lower in the 2% JBGC group than in the control (P < 0.01), whereas no significant differences were observed in the 6% and 10% groups. TNF-α was significantly reduced only in the 10% JBGC group at day 28 (*P* < *0.01*). IFN-γ exhibited a biphasic response: it was significantly elevated in the 10% JBGC group at day 28 (*P* < *0.01*) but significantly reduced in the 6% and 10% groups at day 70 (*P* < *0.01*). Overall, 10% JBGC produced the most pronounced anti-inflammatory profile, and all doses persistently suppressed IL-8.

**Table 8:** Effects of different levels of JBGC in the diet on serum anti-/pro-inflammatory factors of geese.

Indicator	0% JBGC	2% JBGC	6% JBGC	10% JBGC	SEM	*P*-value
IL-1β (pg/ml)						
Day 14	829.32	767.61	721.59	795.79	22.80	0.25
Day 28	711.63	643.33	648.83	648.51	16.23	0.08
Day 70	890.33^a^	900.60^a^	875.49^a^	664.92^b^	56.21	< 0.01
IL-6 (pg/ml)						
Day 14	71.98	78.54	73.61	79.67	1.87	0.27
Day 28	142.03	141.09	149.54	137.68	2.50	0.06
Day 70	111.29^a^	90.48^b^	108.51^a^	119.54^a^	6.12	< 0.01
IL-8 (pg/ml)						
Day 14	31.72	30.77	29.09	28.48	0.75	0.39
Day 28	75.75^a^	64.78^b^	64.95^b^	58.29^b^	3.62	< 0.01
Day 70	54.88^a^	48.92^b^	47.37^b^	41.72^b^	2.71	< 0.01
IL-10 (pg/ml)						
Day 14	122.99	125.35	124.26	129.33	1.37	0.77
Day 28	156.38	147.60	144.06	140.15	3.46	0.59
Day 70	91.94^c^	98.45^bc^	103.14^ab^	110.10^a^	3.83	< 0.01
TNFα (pg/ml)						
Day 14	366.30	352.35	315.44	350.40	10.82	0.11
Day 28	375.12^a^	360.20^a^	354.55^a^	276.32^b^	22.17	< 0.01
Day 70	153.76	151.21	157.96	159.15	1.84	0.35
IFN-γ (pg/ml)						
Day 14	798.52	784.58	697.77	761.07	22.29	0.09
Day 28	649.73^b^	669.10^b^	700.49^ab^	730.81^a^	17.82	< 0.01
Day 70	496.88^a^	479.38^a^	395.11^b^	318.78^c^	41.11	< 0.01

IL, interleukin; TNF-α, tumor necrosis factor-alpha; IFN-γ, interferon-gamma; Ig, immunoglobulin. Data are expressed as mean ± SEM (*n* = 8). Different superscript letters (a, b) within the same row indicate *P* < *0.05*; *P* < *0.01* is indicated by double asterisks where applicable.

### 3.3 Effects of JBGC on serum immunoglobulin concentrations

Serum immunoglobulin levels are presented in Table 9. At 28 days, serum IgA showed a numerical but non-significant increase with increasing JBGC levels. Serum IgM was significantly higher in the 10% JBGC group than in the control at 28 days (*P* < *0.05*), and at 70 days, both the 6% and 10% JBGC groups exhibited significantly higher IgM levels than the control (*P* < *0.05*). No significant differences in serum IgG or other immunoglobulins were detected between any JBGC-supplemented group and the control at any sampling time.

**Table 9:** Effects of different levels of JBGC in the diet on serum immunoglobulin indices of geese.

Indicator	0% JBGC	2% JBGC	6% JBGC	10% JBGC	SEM	*P*-value
IgG pg/ml						
Day 14	49.92	50.10	51.94	52.79	0.70	0.77
Day 28	84.22	82.24	84.92	86.59	0.90	0.28
Day 70	84.31	78.55	80.38	83.53	1.35	0.14
IgA pg/ml						
Day 14	378.58	347.33	326.94	361.43	10.94	0.39
Day 28	426.80^ab^	407.38^b^	446.45^ab^	457.30^a^	11.02	0.02
Day 70	351.39	327.74	334.85	341.30	5.02	0.15
IgM pg/ml						
Day 14	5.29	5.05	4.67	5.21	0.14	0.10
Day 28	6.89^b^	6.88^b^	7.37^b^	7.94^a^	0.25	< 0.01
Day 70	3.23^c^	3.36^c^	3.65^b^	3.94^a^	0.16	< 0.01

IL, interleukin; TNF-α, tumor necrosis factor-alpha; IFN-γ:,interferon-gamma; Ig, immunoglobulin. Data are expressed as mean ± SEM (*n* = 8). Different superscript letters (a, b) within the same row indicate *P* < *0.05*; *P* < *0.01* is indicated by double asterisks where applicable.

### 3.4 Effects of JBGC on small intestinal development

The effects of JBGC on small intestinal development at 70 days of age are shown in Table 10. Significant differences among groups were observed in duodenal length, duodenal weight, jejunal weight, and total small intestinal weight (*P* < *0.05*). Specifically, the 10% JBGC group exhibited significantly greater duodenal length and weight than the 2% JBGC group (*P* < *0.05*). Both the 6% and 10% JBGC groups had significantly higher jejunal weight than the 2% JBGC group (*P* < *0.05*). Total small intestinal weight was significantly greater in the 6% JBGC group than in the 2% JBGC group (*P* < *0.05*). Although jejunal length (81.55 cm vs. 76.20 cm) and total small intestinal length (192.56 cm vs. 184.15 cm) were numerically greater in the 10% JBGC group than in the control, these differences were not statistically significant.

**Table 10:** Effects of different dietary levels of JBGC on small intestinal length and weight of geese.

Index	0% JBGC	2% JBGC	6% JBGC	10% JBGC	SEM	*P*-value
Duodenal length (cm)	34.95^ab^	33.75^b^	33.44^b^	36.78^a^	0.46	0.04
Duodenal weight (g)	8.26^ab^	7.20^b^	9.02^a^	9.01^a^	0.25	0.02
Jejunal length (cm)	76.20	76.14	77.34	81.55	1.03	0.19
Jejunal weight (g)	17.30^bc^	16.04^c^	21.01^a^	19.29^ab^	0.52	< 0.01
Ileal length (cm)	73.00	71.56	70.40	73.54	1.00	0.72
Ileal weight (g)	14.40	13.13	15.51	15.52	0.42	0.12
Total small intestinal length (cm)	184.15	183.92	183.82	192.56	1.93	0.27
Total small intestinal weight (g)	40.45	39.76^b^	45.13	44.58	0.91	0.07

VH, villus height; CD, crypt depth; V/C, ratio of villus height to crypt depth. Values are mean ± SEM (*n* = 8). Superscript letters (a, b) in the same row denote significant difference (*P* < *0.05*).

**Figure 1:**
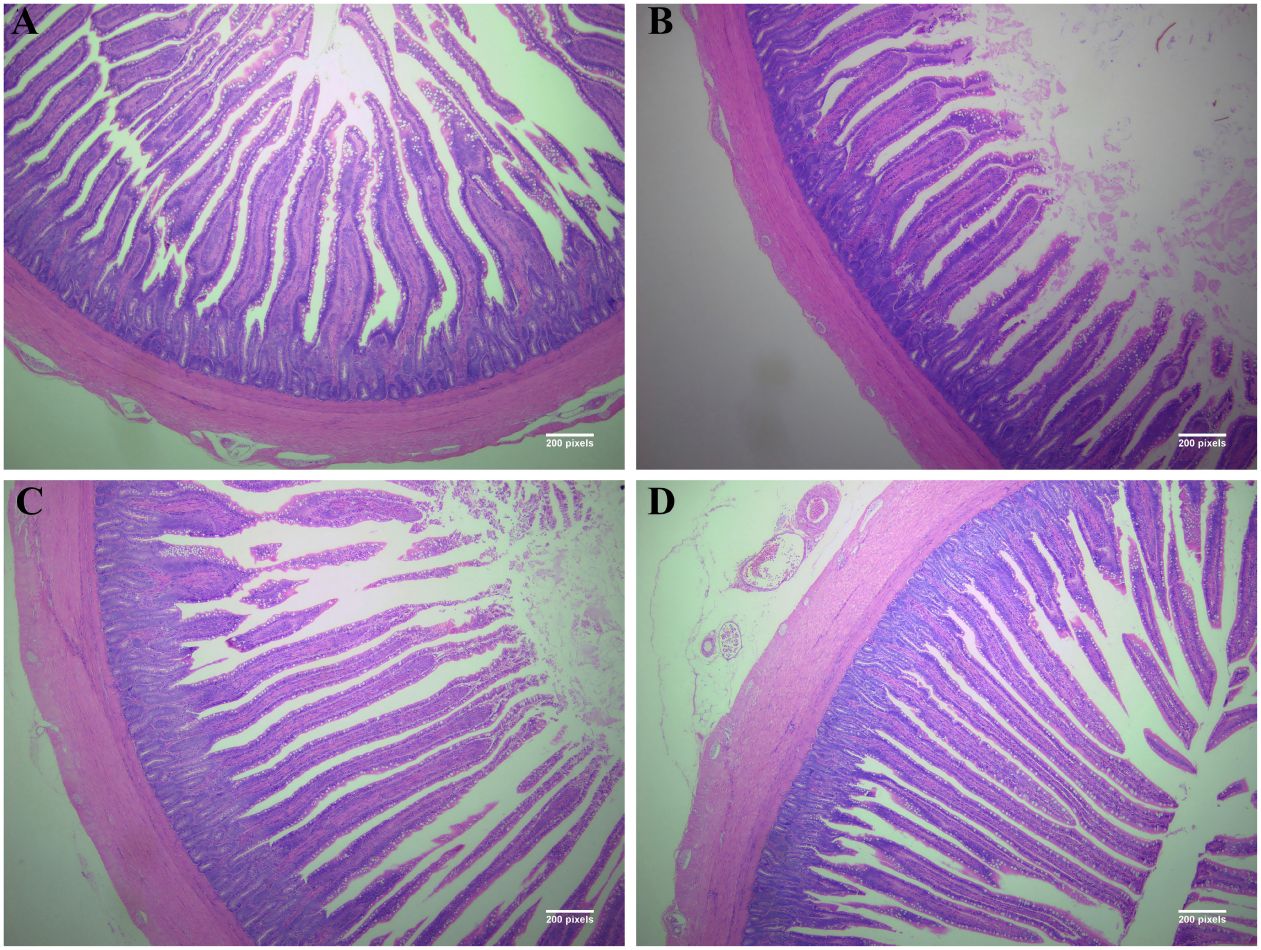
Effects of dietary supplementation with different levels of fermented sauce-flavored distiller's grains on the intestinal tissues of geese (40 × ). A, B, C, D represent HE-stained histological sections of the 0% JBGC group **(A)**, 2% JBGC group **(B)**, 6% JBGC group **(C)**, and 10% JBGC group **(D)**, respectively.

Ileal morphological parameters are summarized in Figure 1 and Table 11. Compared with the control, villus height was significantly lower in the 2% JBGC group (*P* < *0.05*) but significantly higher in the 6% and 10% JBGC groups (*P* < *0.05*). No significant differences were observed in crypt depth, V/C ratio, or muscular layer thickness between any JBGC-supplemented group and the control, and overall intestinal morphology remained normal across all groups.

**Table 11:** Effects of different dietary levels of JBGC on intestinal villus morphology of geese.

Indices (μm)	0% JBGC	2% JBGC	6% JBGC	10% JBGC	SEM	*P*-value
Villus height	981.16^b^	795.22^c^	1,006.97^ab^	1,122.85^a^	67.85	0.01
Villus width	111.82	129.91	111.37	123.91	4.588	0.53
Crypt depth	264.32	226.67	275.60	249.11	10.58	0.07
Muscular layer depth	276.44	273.94	309.44	272.37	8.84	0.49
Villus height/crypt depth ratio (V/C ratio)	3.77	3.54	3.78	4.53	0.22	0.18

VH, villus height; CD, crypt depth; V/C, ratio of villus height to crypt depth. Values are mean ± SEM (*n* = 8). Superscript letters (a, b) in the same row denote significant difference (*P* < 0.05).

### 3.5 Effects of JBGC on the expression of intestinal barrier function-related genes and proteins

**Figure 2:**
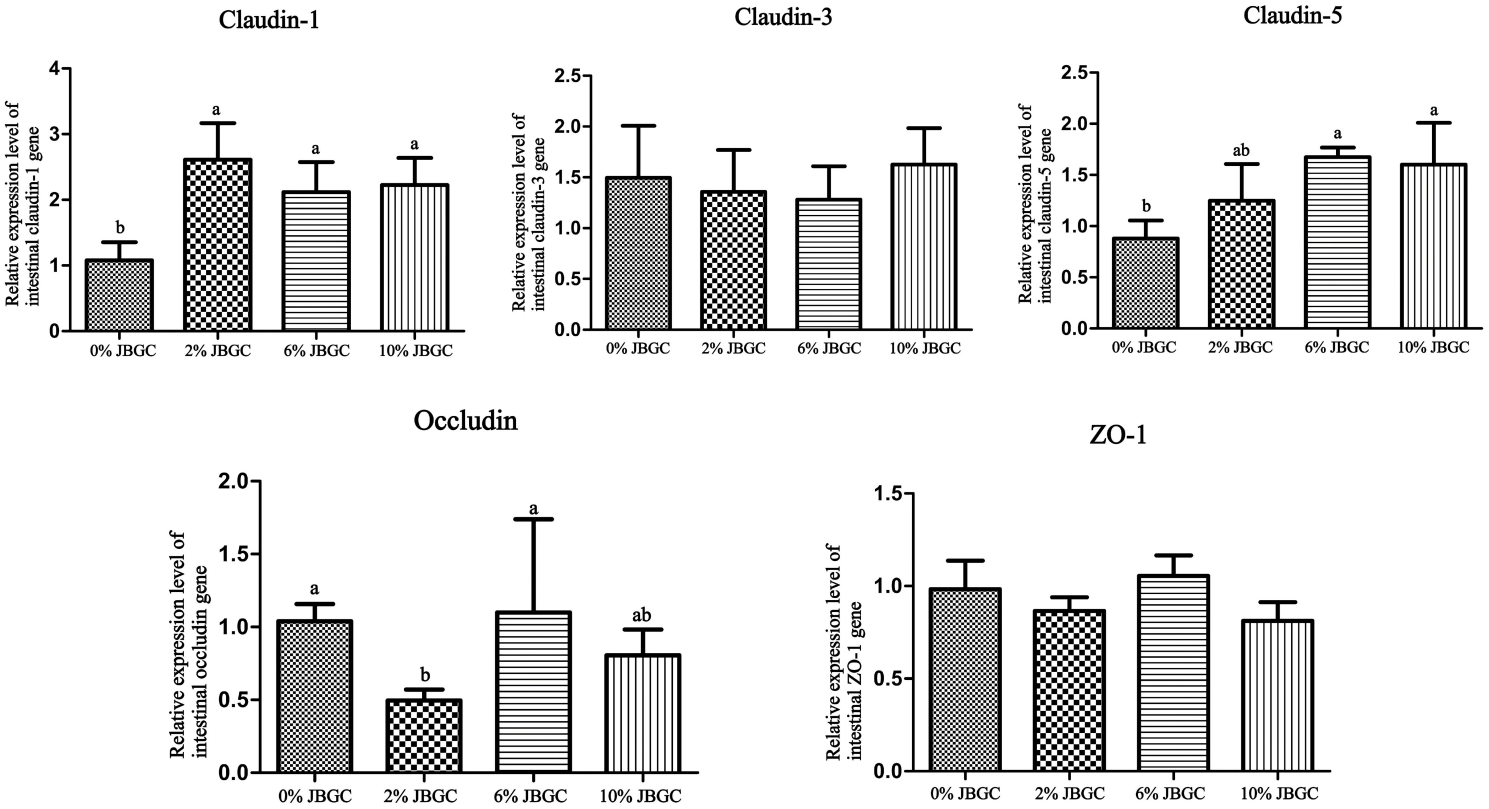
Effects of different dietary levels of JBGC on the expression of intestinal barrier function-related genes in geese.

The effects of JBGC on mRNA expression of tight junction genes (*occludin, claudin-1, claudin-3, claudin-5, ZO-1*) in the ileum are shown in Figure 2. *Claudin-1* expression was significantly upregulated in all JBGC-supplemented groups compared with the control (*P* < 0.05). *Claudin-5* expression was significantly higher in the 6% and 10% JBGC groups than in the control (*P* < *0.05*). Occludin expression was significantly lower in the 2% JBGC group than in the control (*P* < *0.05*). No significant differences were observed in *claudin-3* or *ZO-1* expression between any JBGC-supplemented group and the control.

**Figure 3:**
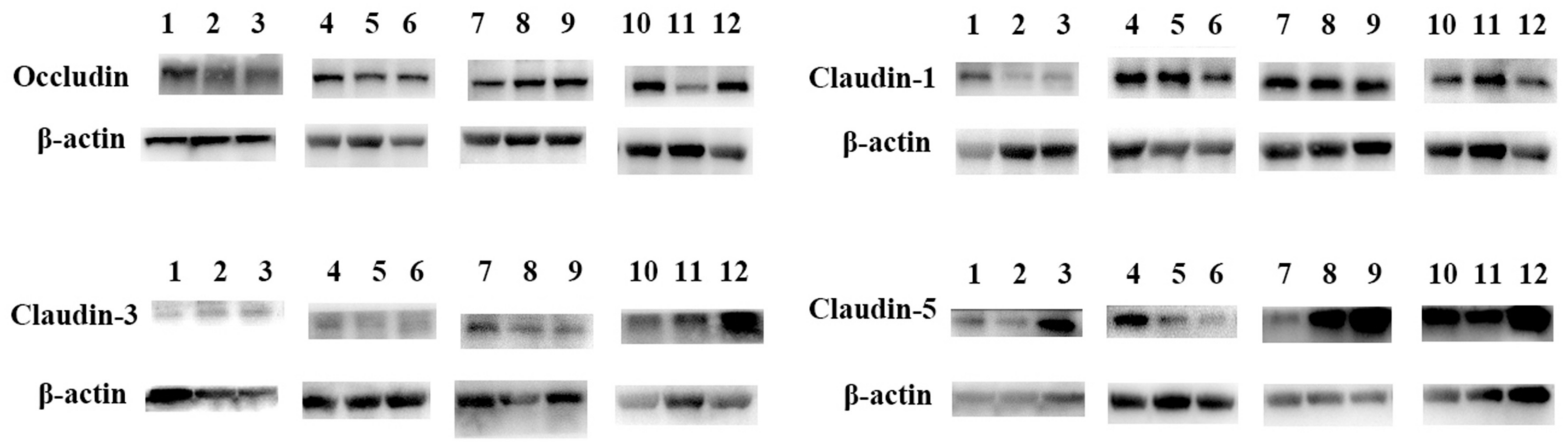
Expression of intestinal barriier function-associated proteins in geese. 0% JBGC Group (1–3); 2% JBGC Group (4–6); 6% JBGC Group (7–9); 10% JBGC Group (10–12).

**Figure 4:**
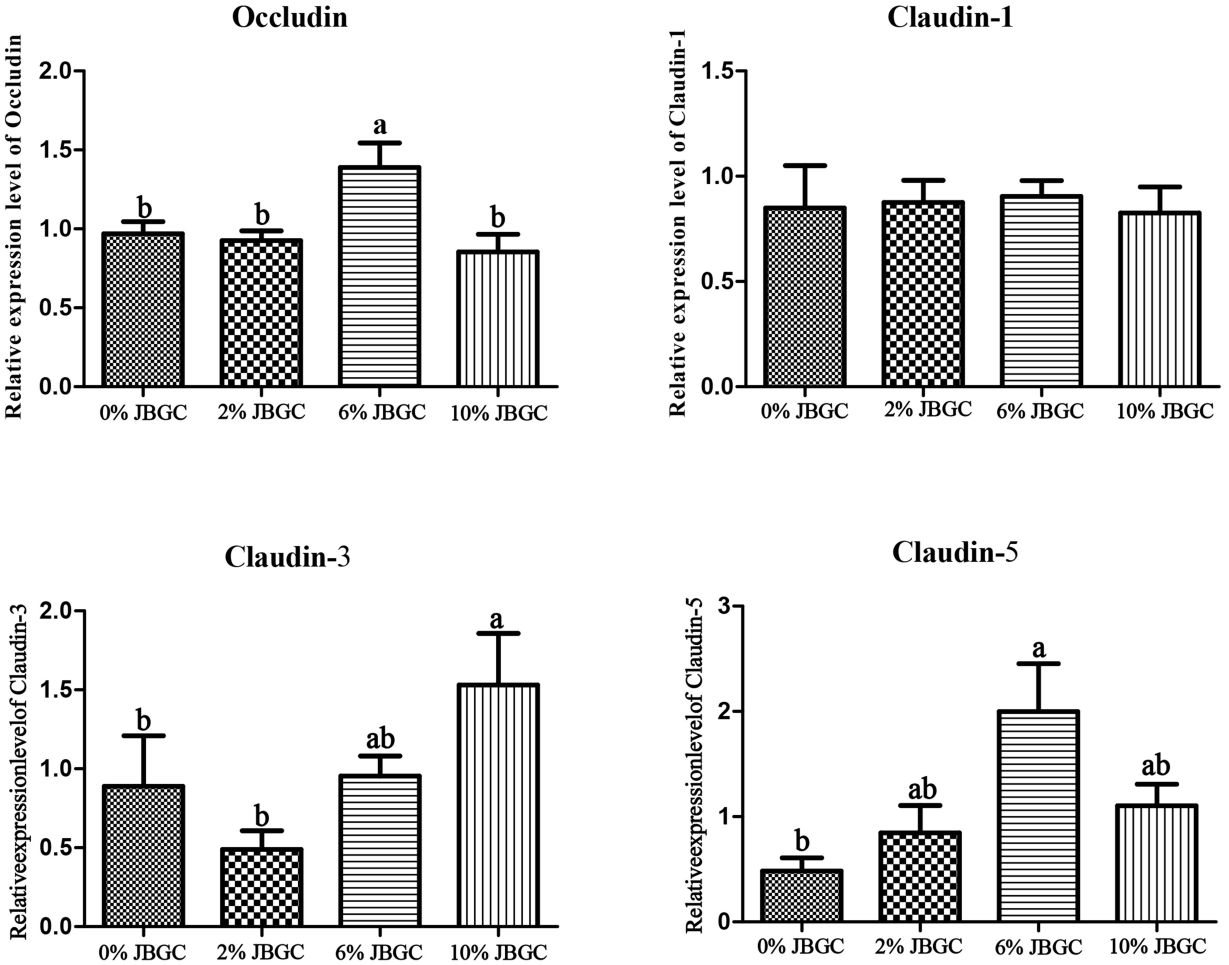
Densitometric analysis of intestinal barrier-associated protein expression in geese.

Protein expression levels of tight junction proteins are presented in Figures 3, 4. Compared with the control, occludin and claudin-5 protein levels were significantly higher in the 6% JBGC group (*P* < *0.05*), and claudin-3 protein level was significantly higher in the 10% JBGC group (*P* < *0.05*). No significant differences were detected for other proteins.

### 3.6 Effects of JBGC on ileal cytokine gene expression

**Figure 5:**
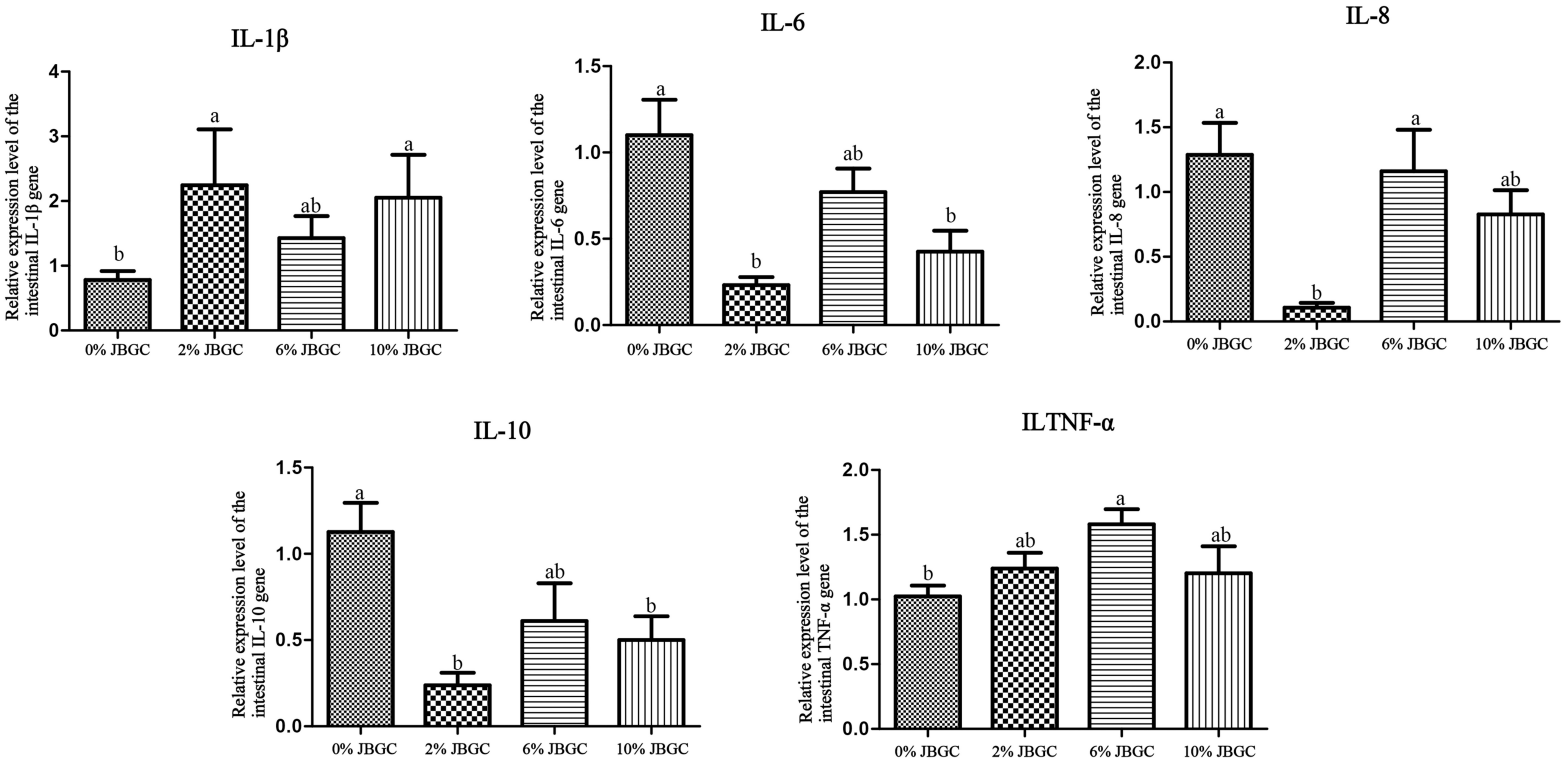
Effects of JBGC on the gene expression of intestinal pro-inflammatory and anti-inflammatory factors.

The effects of JBGC on ileal mRNA expression of inflammatory cytokines (IL-1β, IL-6, IL-8, IL-10, TNF-α) are shown in Figure 5. Compared with the control, the 2% JBGC group exhibited significantly higher IL-1β expression and significantly lower expression of IL-6, IL-8, and IL-10 (*P* < *0.05*). The 6% JBGC group showed significantly higher TNF-α expression (*P* < *0.05*). The 10% JBGC group exhibited significantly higher IL-1β expression and significantly lower IL-6 and IL-10 expression (*P* < *0.05*). No other significant differences were observed.

### 3.7 Effects of JBGC on cecal microbiota composition

- Effects on intestinal microbiota diversity

**Figure 6:**
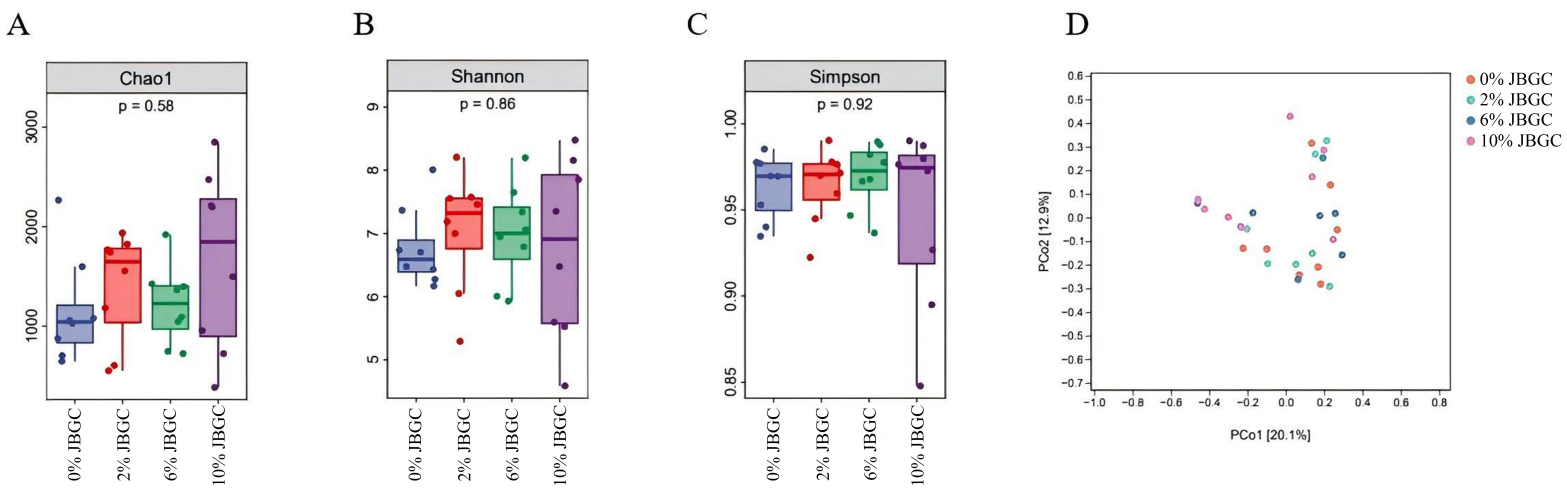
**(A–D)** Assessment of alpha and beta diversity in cecal microbial communities.

Alpha diversity indices (Chao1, Shannon, Simpson) of the cecal microbiota are shown in Figures 6A–C. Although Chao1 and Shannon indices were numerically higher in JBGC-supplemented groups than in the control, these differences were not statistically significant (*P* > *0.05*). Principal coordinate analysis (PCoA) based on Bray–Curtis distances (Figure 6D) revealed no distinct clustering of microbial communities among treatment groups, indicating similar overall composition.

- Effects on intestinal microbial community structure

**Figure 7:**
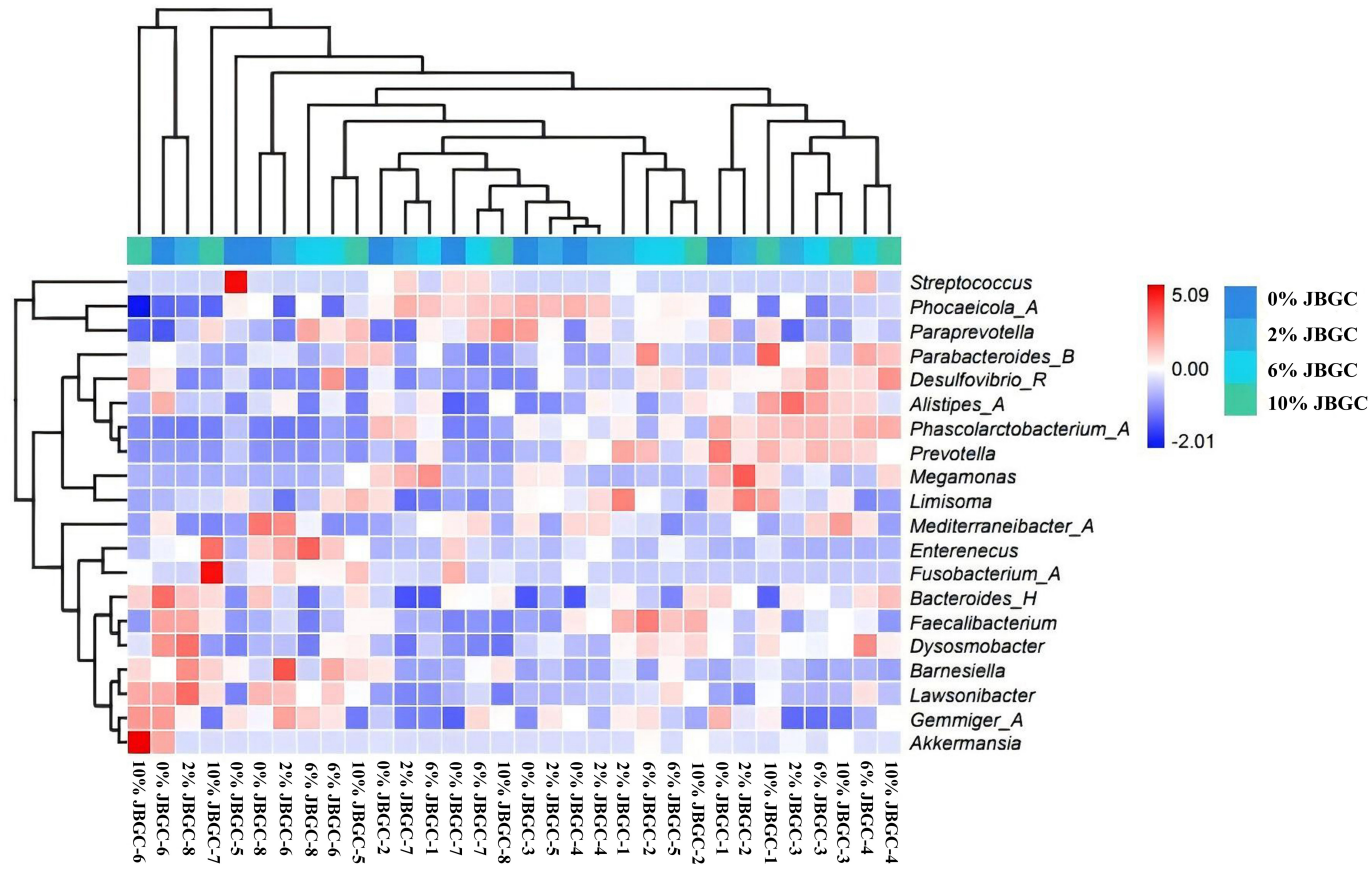
Effects of dietary fermented JBGC at varying levels on cecal genus-level microbial diversity in geese: A Heatmap analysis.

At the genus level, no significant differences in relative abundance were detected among groups (*P* > *0.05*; Figure 7, Table 12). However, numerical trends were observed: JBGC-supplemented groups showed increased abundances of *Paraprevotella, Desulfovibrio_R*, and *Barnesiella*, and decreased abundances of *Phocaeicola_A, Streptococcus*, and *Mediterraneibacter_A* compared with the control.

**Table 12:** Effects of dietary supplementation with different levels of fermented JBGC on the composition of cecal microbiota at the genus level (%).

Genus level	0% JBGC	2% JBGC	6% JBGC	10% JBGC	SEM	*P*-value
Phocaeicola_A	37.88	36.32	33.80	25.87	2.66	0.40
Bacteroides_H	4.89	4.29	3.40	5.43	0.44	0.41
Paraprevotella	3.65	2.50	5.58	5.15	0.55	0.17
Desulfovibrio_R	2.70	2.58	5.95	4.70	0.70	0.26
Barnesiella	2.50	5.69	3.14	4.44	0.85	0.57
Enterenecus	3.20	2.94	4.90	3.84	1.03	0.92
Phascolarctobacterium_A	3.20	3.52	3.22	3.13	0.52	0.99
Faecalibacterium	2.60	2.93	2.96	2.81	0.48	0.99
Megamonas	2.83	4.07	2.20	2.04	0.77	0.80
Lawsonibacter	2.38	2.82	2.78	2.77	0.45	0.99
Prevotella	2.12	2.37	2.79	2.26	0.47	0.97
Parabacteroides_B	1.37	1.31	2.29	2.35	0.27	0.36
Gemmiger_A	1.80	1.61	1.59	1.46	0.17	0.91
Akkermansia	1.55	0.20	0.37	4.28	1.00	0.46
Alistipes_A	1.17	1.64	1.39	1.61	0.18	0.80
Fusobacterium_A	1.32	0.69	0.58	3.13	0.59	0.41
Dysosmobacter	0.83	1.05	1.15	0.99	0.16	0.92
Limisoma	1.00	1.30	0.64	1.01	0.14	0.47
Streptococcus	2.23	0.51	0.98	0.05	0.52	0.38
Mediterraneibacter_A	1.11	0.86	0.81	0.49	0.14	0.50

Values are mean relative abundance (%) ± SEM (*n* = 8). Superscript letters (a, b) indicate significant difference (*P* < *0.05*) by one-way ANOVA. No superscript letters indicate no significant difference among groups.

At the phylum level, the cecal microbiota across all treatment groups was dominated by *Firmicutes, Bacteroidota*, and *Proteobacteria*, which collectively accounted for >95% of total sequences. No significant differences in the relative abundances of these phyla were observed among the 0%, 2%, 6%, and 10% JBGC groups (*P* > *0.05*). Similarly, at the family level, *Ruminococcaceae, Lachnospiraceae*, and *Bacteroidaceae* were the most abundant families, and their relative abundances did not differ significantly among treatments (*P* > *0.05*). A full list of family-level abundances is provided in the genus-level data (Table 12), as family membership can be inferred from the listed genera.

## 4 Discussion

This study systematically investigated the regulatory effects of dietary JBGC on immune function, oxidative stress, and intestinal health in Magang geese, with emphasis on dose-dependent mechanisms. As a complex fermented product rich in yeast β-glucan, SCFA precursors, and functional proteins, JBGC exerted multi-targeted regulation via interactions with immune signaling pathways, oxidative stress cascades, and the gut microbiota–barrier axis. These effects aligned with the known biological properties of yeast-derived additives while also reflecting species-specific responses in waterfowl. Consistent with recent findings in geese (19), JBGC supplementation improved intestinal morphology and digestive enzyme activities, further validating the species-specific benefits observed in our study.

The immunomodulatory effects of JBGC were dose dependent, likely reflecting differential engagement of immune signaling pathways by its bioactive components. At 10% inclusion, serum IL-10 was significantly elevated, whereas IL-1β was moderately suppressed (Table 8). This cytokine profile—elevated IL-10 with restrained IL-1β–is consistent with macrophage modulation by yeast β-glucan, which promotes a regulatory phenotype that curbs excessive inflammation while preserving microbial clearance capacity, thereby avoiding immune paralysis (20). Regarding the temporal dynamics observed in Table 8 and Table 9 (control group), the continuous rise in IL-1β from day 14 to day 70 likely reflects its dual role in homeostatic immune surveillance and tissue growth, which remain active throughout the grower-finisher period. In contrast, the peak at day 28 followed by a decline at day 70 for IL-6, IL-8, IL-10, TNF-α, IFN-γ, and all three immunoglobulins (IgA, IgM, IgG) indicates a transient immune activation during the early growth phase (around the time of dietary transition and gut microbiota establishment), followed by the establishment of immune homeostasis by day 70. Importantly, JBGC supplementation did not disrupt this physiological trajectory but rather modulated the magnitude of responses, exerting its most pronounced anti-inflammatory effects at day 70. Furthermore, 10% JBGC specifically increased serum IgM (Table 9) without significantly affecting IgG or IgA. This pattern aligned with the role of IgM as an early responder in waterfowl humoral immunity. In geese, the 70-day observation period may be insufficient to capture later-phase immunoglobulin (IgG/IgA) responses, which typically require over 90 days for full maturation of secondary lymphoid organs (21). This developmental trajectory differed from that of faster-maturing broilers, in which yeast culture upregulates both IgG and IgA within shorter periods (13, 22). Notably, the 2% JBGC dose elicited limited or neutral effects on many immune parameters, suggesting a sub-threshold stimulus for systemic immune modulation—adequate perhaps for local intestinal interactions but insufficient to drive a robust systemic response, or indicative of an early adaptive phase. The bidirectional regulation of IL-6 (lower in the 2% group, normalized at 6% and 10%) further illustrated the dose-sensitive nature of immune tuning. Reduced IL-6 at 2% JBGC may reflect lower initial immune stimulation or a muted inflammatory tone, possibly linked to the modest reduction in villus height (Table 11). In contrast, normalization of IL-6 at higher doses suggested a state of balanced “defensive readiness,” wherein adequate immune activation was achieved without overt inflammation—a response pattern previously associated with well-modulated T-helper cell activity (23).

The antioxidant effects of JBGC also exhibited a clear dose threshold (6%−10% effective; 2% adverse, e.g., reduced hepatic T-AOC; Table 7), attributable to substrate availability and efficiency of Nrf2 pathway activation. Yeast β-glucan and fermented metabolites (e.g., glutathione precursors) in JBGC activate hepatic Nrf2, promoting transcription of antioxidant enzymes (T-SOD, GPx). The 10% JBGC likely provided sufficient substrates for full Nrf2 activation, increasing hepatic T-SOD and serum GPx (Table 6), whereas 2% JBGC supplied inadequate precursors and may induce mild hepatic metabolic stress due to limited yeast cell wall components, thereby reducing T-AOC. This threshold effect aligned with findings in selenium yeast studies (24), where 0.3 mg/kg was optimal and < 0.1 mg/kg ineffective, suggesting a universal dose threshold for yeast-derived additives. Additionally, 10% JBGC elevated serum T-AOC at both 28 and 70 days (Table 6), indicating sustained antioxidant capacity—unlike rapidly metabolized synthetic antioxidants (e.g., vitamin E), JBGC-derived SCFAs and β-glucan may provide continuous stimulation of the antioxidant system throughout the grower–finisher stages. The biphasic temporal pattern of serum T-AOC (decrease at day 28, increase at day 70) likely results from an initial adaptive phase to the novel dietary intervention, followed by sustained upregulation of antioxidant pathways. Early reduction may reflect metabolic reallocation or mild oxidative stress induced by the yeast culture, whereas the later elevation indicates successful activation of the Nrf2-mediated antioxidant response after prolonged exposure.

JBGC promoted intestinal health via a dual mechanism: direct stimulation of epithelial function and indirect modulation of the microbiota. First, 6%−10% JBGC significantly increased ileal villus height (Table 11), likely linked to SCFA production. Fermentable fiber in JBGC (e.g., distiller's grain cellulose) is degraded by cecal microbiota (e.g., *Paraprevotella*; Table 12) to yield SCFAs, particularly butyrate, which activates intestinal epithelial GPR43, promoting proliferation and inhibiting apoptosis (25), thereby increasing villus height and absorptive surface area. Similar improvements in intestinal morphology and barrier function have been reported in aged laying hens fed yeast culture (26), supporting the translational potential of JBGC across different poultry species and production stages. In contrast, the lower fiber content in 2% JBGC likely resulted in insufficient SCFA production, and residual yeast components may compete with epithelial cells for nutrients, leading to reduced villus height relative to the control. Second, JBGC upregulated tight junction proteins (occludin, claudin-3/5; Table 12). Discrepancies between gene and protein expression (e.g., lower occludin mRNA in 2% JBGC but higher occludin protein in 6% JBGC) suggested post-translational regulation. Occludin stability is modulated by phosphorylation (27); JBGC-derived SCFAs can activate protein kinase C (PKC), promoting occludin phosphorylation and reducing degradation. This may explain the elevated occludin protein at 6% JBGC despite unchanged mRNA levels, underscoring the importance of multi-level (transcriptional and translational) assessment of barrier function.

Although the 70-day JBGC intervention did not significantly alter overall cecal microbial α-diversity or genus-level structure—potentially due to insufficient dosage or duration, host-specific factors, or high inter-individual variation—it appeared to induce functional remodeling rather than structural disruption. Notably, numerical increases in *Paraprevotella* (a butyrate producer) and decreases in *Streptococcus* (a potential pathogen) were observed. Butyrate derived from *Paraprevotella* can promote villus growth and lower intestinal pH, thereby inhibiting *Streptococcus* colonization and reducing epithelial invasion risk (28). Furthermore, butyrate is also known to directly upregulate the expression of tight junction proteins, including occludin and claudins, via activation of AMPK or HDAC inhibition, which aligns with our observation that 6% JBGC increased occludin and claudin-5 protein levels (Figures 3, 4). This interaction reinforces the intestinal barrier and supports the concept of a “microbiota–metabolite–barrier” positive feedback loop.

Consistent with recent findings in broilers fed distiller's grain-based yeast cultures (29, 30), our study observed a shift in microbial composition toward a more beneficial profile, suggesting that this functional feed additive exerts conserved modulatory effects on gut microbiota across avian species. Additionally, the numerically lower abundance of *Phocaeicola_A* in JBGC groups, though not significant, could suggest a shift away from certain SCFA-producing pathways. However, the concurrent increase in *Paraprevotella* and *Barnesiella* (both also SCFA producers) indicates functional redundancy; overall microbial fermentation capacity for SCFA production is likely preserved or enhanced, consistent with the beneficial intestinal morphological changes observed. The absence of significant treatment effects at the phylum and family levels, despite numerical changes at the genus level (e.g., increased *Paraprevotella*, decreased *Streptococcus*), is likely explained by functional redundancy among members within the same higher taxonomic ranks. For example, multiple genera within the phylum Firmicutes can produce butyrate, and a decrease in one genus may be compensated by an increase in another (as observed for *Phocaeicola_A* vs. *Paraprevotella*). Additionally, the overall supplementation period (70 days) and the relatively low inclusion levels (2%−10%) may have been insufficient to drive community-wide restructuring at broader taxonomic levels. These observations are consistent with the concept that dietary interventions often induce subtle, genus-specific shifts rather than dramatic alterations in phylum or family composition.

This study had several limitations. First, the 70-day observation period focused on grower–finisher stages; long-term effects (e.g., during laying) on egg quality and immune memory remain unknown. Second, the complex composition of JBGC precluded definitive attribution of observed effects to individual components (e.g., yeast β-glucan, SCFA precursors, functional peptides); future studies using purified components are needed. Third, analyses were confined to blood, liver, and intestine; immune organs (e.g., spleen, bursa of Fabricius) and their cytokine secretion patterns were not examined, limiting our understanding of systemic immune regulation.

## 5 Conclusions

Dietary supplementation with fermented Jiang-flavor distiller's grain-derived yeast culture (JBGC) improved physiological health in Magang geese through a multi-pathway, dose-dependent network, with 6%−10% representing the optimal inclusion range. Immunologically, JBGC increased IgM (early humoral immunity) and IL-10 (anti-inflammatory response) while bidirectionally regulated IL-6 to balance defense and metabolic demands. Antioxidantly, high-dose JBGC (10%) enhanced hepatic and serum antioxidant enzyme (T-SOD) activity, whereas the low dose (2%) does not elicit this response. In the intestine, JBGC promoted villus proliferation and tight-junction stabilization, and optimized the microbiota–barrier axis by increasing *Paraprevotella* and reducing *Streptococcus*. These findings support the application of JBGC in waterfowl feeding and underscore the importance of dose and species specificity in feed additive evaluation. Future research should focus on isolating the active components of JBGC and assessing its long-term effects to fully realize its potential as an antibiotic alternative in sustainable poultry production.

## Data Availability

The original contributions presented in the study are included in the article/supplementary material, further inquiries can be directed to the corresponding authors.
